# Power Estimation for Gene-Longevity Association Analysis Using Concordant Twins

**DOI:** 10.1155/2014/154204

**Published:** 2014-09-16

**Authors:** Qihua Tan, Jing Hua Zhao, Torben Kruse, Kaare Christensen

**Affiliations:** ^1^Epidemiology, Biostatistics and Biodemography, Institute of Public Health, University of Southern Denmark, J. B. Winsløws Vej 9B, 5000 Odense C, Denmark; ^2^Department of Clinical Genetics, Odense University Hospital, 5000 Odense C, Denmark; ^3^MRC Epidemiology Unit, University of Cambridge School of Clinical Medicine, Cambridge CB2 0QQ, UK

## Abstract

Statistical power is one of the major concerns in genetic association studies. Related individuals such as twins are valuable samples for genetic studies because of their genetic relatedness. Phenotype similarity in twin pairs provides evidence of genetic control over the phenotype variation in a population. The genetic association study on human longevity, a complex trait that is under control of both genetic and environmental factors, has been confronted by the small sample sizes of longevity subjects which limit statistical power. Twin pairs concordant for longevity have increased probability for carrying beneficial genes and thus are useful samples for gene-longevity association analysis. We conducted a computer simulation to estimate the power of association study using longevity concordant twin pairs. We observed remarkable power increases in using singletons from longevity concordant twin pairs as cases in comparison with cases of sporadic proband. A similar power would require doubled sample sizes for fraternal twins than for identical twins who are concordant for longevity suggesting that longevity concordant identical twins are more efficient samples than fraternal twins. We also observed an approximate of 2- to 3-fold increase in sample sizes needed for longevity cutoff at age 90 as compared with that at age 95. Overall, our results showed high value of twins in genetic association studies on human longevity.

## 1. Introduction

Complex phenotypes such as human longevity are associated with multiple genetic and environmental factors with perhaps the majority of them having low to modest effects [1]. As such, the power issue has been a crucial concern in genetic association studies. Although a desired statistical power can always be achieved by increased sample sizes, there can be many factors including the laboratory cost that easily limit the scale of a study. This is especially true for the currently still expensive genomic analysis, for example, the genome sequencing technologies. Twins are special samples that have made remarkable contribution to human genetic studies due to their genetic and environmental sharing. In genetic epidemiology, the popular classical twin design has been widely used in estimating the genetic and environmental components in the variation of disease phenotypes or traits [2]. For example, using Danish twins, the genetic contribution to human longevity has been estimated as about 25% [3, 4]. The low heritability and the complex nature of human longevity make genetic association study on the trait low powered. In the literature, the search for genes associated with longevity has continued over many decades with only one gene, APOE, being conclusively confirmed.

Because of their genetic relatedness, twin pairs concordant for longevity are enriched for carrying beneficial genes and thus association studies using singletons from longevity concordant twin pairs should have increased power in comparison with using sporadic longevity individuals. This paper assesses and explores the power advantage for the use of longevity concordant twin pairs by computer simulation. The simulation is based on a proportional hazard assumption and makes use of the recent life table data of the Danish population. Lifespan data will be generated for identical or monozygotic (MZ) and fraternal or dizygotic (DZ) twin pairs with power compared between zygosities and across different experiment setups.

## 2. Materials and Methods

### 2.1. Experiment Design

**Figure 1:**
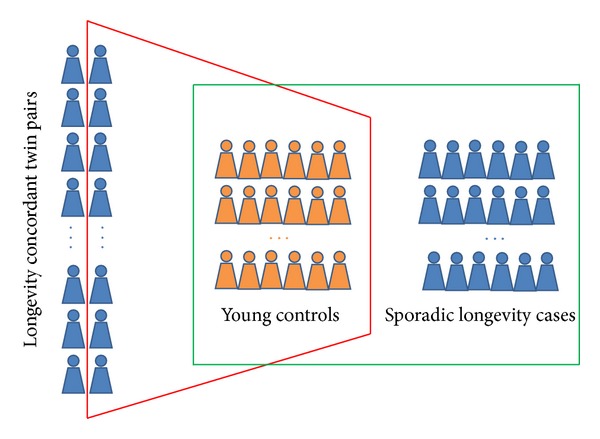
The concordant design and the ordinary case-control design for gene-longevity association study. The red frame represents concordant twin design in which singletons from longevity twin pairs (blue) are collected as cases. In contrast, the red frame is the ordinary case-control design in which sporadic longevity cases (blue) are sampled. Both designs use young subjects in the middle (orange) as controls.

The most popular experiment design for genetic association study on human longevity is the case-control design which samples longevity individuals (e.g., centenarians and nonagenarians) as cases and young or middle aged individuals as controls [5]. The power issue for the case-control design has been investigated by Tan et al. [6]. The current simulation study focuses on the power advantage of using singleton twins from twin pairs concordant for longevity as cases (Figure 1). That is, from each concordant twin pair reaching certain threshold for longevity (e.g., 90 or 95 in this simulation), only one twin will be taken as case for genotyping. The controls will be collected as in ordinary case-control studies from unrelated individuals. With this design, the final data for analysis contain unrelated cases and controls with cases collected as singletons from longevity concordant twin pairs (one from each pair) and controls as unrelated individuals at age 40–50 years. The study design draws equal number of cases and controls in the final sample.

### 2.2. The Danish Life Table Data

Our simulation of individual lifespan data is based on Danish population survival information, that is, the Danish life table data available from Statistics Denmark (http://www.statbank.dk/). The current simulation work used the most recent Danish life table data for period 2012-2013 with life expectancy 78 years for males and 82 years for females. The simulation takes the mean survival rate over the two sexes. The use of observed population survival rate avoids imposing any parametric form for the survival function in the simulation and thus ensures that our simulated survival data follows survival distribution in a real population (i.e., the Danish population).

### 2.3. The Proportional Hazard Model

For a given genetic variant, for example, single nucleotide polymorphism (SNP), we assign a frequency parameter *p* and a relative risk parameter *r* for the minor allele of the SNP. Then the observed population survival rate at any age *x* is a weighted average of three subpopulations carrying 0, 1, and 2 copies of the minor allele, respectively [7], that is,

(1) $$\begin{array}{c} \bar{s}\left(x\right)=p^{2}s_{2}\left(x\right)+2p\left(1-p\right)s_{1}\left(x\right)+{\left(1-p\right)}^{2}s_{0}\left(x\right). \end{array}$$

In (1), $\bar{s}\left(x\right)$, *s*_0_(*x*), *s*_1_(*x*), *s*_2_(*x*) are survival rate for the total population and for the three subpopulations at age *x*; (1−*p*)^2^, 2*p*(1 − *p*), *p* ^2^ are frequencies for corresponding genotypes following binomial distribution of the minor allele frequency (MAF), *p*. With the proportional hazard assumption, the hazards of death corresponding to *s*_1_(*x*) and *s*_2_(*x*) can be expressed as *μ*_1_(*x*) = *rμ*_0_(*x*) and *μ*_2_(*x*) = *r* ^2^ *μ*_0_(*x*) so that

(2) $$\begin{array}{c} s_{1}\left(x\right)=e^{-\int_{0}^{x}\mu_{1}(t)dt}=e^{-\int_{0}^{x}r\mu_{0}(t)dt}={\left(e^{-\int_{0}^{x}\mu_{0}(t)dt}\right)}^{r}=s_{0}{\left(x\right)}^{r}, \end{array}$$

and likewise, *s*_2_(*x*) = *s*_0_(*x*)^*r*^2^^. With these relationships and for given MAF *p* and relative risk *r*, (1) can be solved numerically to obtain a nonparametric estimate of the baseline survival *s*_0_(*x*) and then *s*_1_(*x*) and *s*_2_(*x*) can be calculated and used for generating individual lifespan. In the simulation, we introduced a heterogeneity model for the baseline survival function in order to take into account of the unobserved factors that also affect individual survival [8].

### 2.4. Simulating Lifespan

In order to simulate lifespan using genotype-specific survival, *s*_0_(*x*), *s*_1_(*x*), and *s*_2_(*x*), a genotype will be randomly assigned to each individual using the binomial probability of MAF. For MZ twin pairs, this was first done for one singleton and then the same genotype was copied to the cotwin. For DZ twin pairs, we started with independently simulating genotypes for each parent of a twin pair and assigned genotype for a singleton in a DZ pair by randomly taking one allele from each parent. This ensures that two twins within a pair have 50% chance of inheriting an allele identical by descent (IBD). The lifespan for unrelated controls was simulated by randomly assigning a genotype to each control subject using the binomial distribution of the minor allele. Subjects at age 40–50 years were selected as controls. We simulated power for cases from concordant MZ and DZ twin pairs separately aiming at comparing power difference between zygosities.

### 2.5. Statistical Testing and Power Calculation

Since the samples collected in our simulation design are case-control samples, the popular Armitage's trend test [9] was applied for statistical testing on our simulated samples in each replicate. Similar test had been used in our previous power simulation for genetic association studies on human longevity [6, 10]. Power simulation was done for different combinations of mode of inheritance (additive, dominant, and recessive), allele frequency (MAF = 0.05, 0.1, 0.2, 0.5), and risk of allele (*r* = 0.5, 0.7,0.8,0.85,0.9,0.95) and for different sample sizes of cases (*N* = 100, 200,300,500,800,1500). For each combination, *K* = 1000 replicates were simulated and statistical testing was applied to each replicate. With 1000 *χ* ^2^ values obtained from Armitage's trend test, corresponding power was calculated as

(3) $$\begin{array}{c} \text{Power}\left(\alpha =0.05\right)=\frac{\sum_{i=1}^{K}I\left[\chi^{2}\left(i\right)\geq \chi_{0.05,1}^{2}\right]}{K}, \end{array}$$

where *I*(·) is an indicator function for logical expression with 1 if true and 0 if false.

## 3. Results

In Table 1, we show the power estimates for additive effect of SNP alleles with different combinations of genetic parameters (MAF, relative risk) for different sample sizes from concordant MZ twins and for different cutoffs of longevity. With 800 cases aged 95+, the design is able to identify a common SNP (MAF = 0.5) with a small effect of only 5% reduction of rate of death (*r* = 0.95). For a small sample size of 200 cases aged over 95 years, the study design has good power (over 0.8) to capture a common SNP with MAF = 0.5 that reduces hazard of death by 10% (*r* = 0.9); a SNP with lower MAF = 0.1 and hazard reduction of 15% (*r* = 0.85); and a rare SNP with MAF = 0.05 and hazard reduction of 20% (*r* = 0.8). A small sample of 100 cases aged 95+ is able to detect a common SNP (MAF = 0.2) with risk reduction of 15% (*r* = 0.85). When the longevity cut-off is set to 90 years, a sample size of 500 to 800 cases is required to achieve comparable power, an increase of about 3 folds. The power for detecting dominant effect SNPs using MZ cases (Table 2) is almost comparable with that for the additive effect SNPs with low MAF in Table 1 although the difference increases with increasing MAF. Note that, for dominant effect SNPs, the power starts to decline when MAF approaches 0.5. The statistical power is largely reduced for recessive effect SNPs (Table 3). However, for high MAF SNPs (*p* = 0.5), the design has good power with 500 cases aged over 95 for *r* = 0.9. Comparable power can be achieved with only 200 cases for *r* = 0.85. Interestingly, when comparing power estimates between the two longevity cutoffs (90 and 95 years) for defining cases, we see that the cutoff of 90+ needs 2 to 3 times larger sample sizes to obtain comparable power as compared with that of 95+, a conclusion that applies to Tables 1 to 3.

Tables 4, 5, and 6 carry power estimates for similar parameter settings corresponding to Tables 1–3 except that we added a bigger sample size of 1500 cases considering the relative ease in sampling DZ than MZ concordant twin pairs. The power estimates for DZ twins exhibit similar pattern as for MZ twins but a 2-3-fold increase in sample size is required to obtain comparable power for corresponding settings as in Tables 1–3.

## 4. Discussions

Using computer simulation, we have estimated statistical power for a case-control design using singleton cases from twin pairs concordant for longevity. Different from the ordinary case-control studies that collect sporadic centenarians as cases, we limit our cutoffs for longevity to 90 and 95 considering rarity of twin pairs concordant for extreme longevity. It is interesting to compare our power estimates with those from our previous simulation study on ordinary case-control design with sporadic nontwin centenarians as cases [6]. Even with lowered threshold for longevity at age 95, the concordant MZ twin design is able to achieve equivalent power as in ordinary case-control design with centenarian cases for similar or even smaller sample sizes (comparing Tables 1–3 with Tables 1–3 in Tan et al. [6]). With an age cutoff at 90 years, our power estimates can be compared to those in our previous simulation which also simulated power for using nonagenarians as cases (Tables 4–6 in Tan et al. [6]). For comparable power estimates, the case-control design with cases as sporadic nonagenarians would need much larger sample sizes compared with using nonagenarian cases from concordant twin pairs (3-4 folds for MZ and about 2 folds for DZ twins). Overall, our results revealed remarkable power advantage in using longevity concordant twins over ordinary case-control design.

Comparing the power estimates in Tables 1–3 with those in Tables 4–6, we observe that a power advantage in using cases of MZ twins over cases of DZ twins with the latter requiring almost doubled sample sizes to reach equivalent power. Although relatively lower powered, the DZ twin pairs are actually the same as sibling pairs in genetic sharing meaning that, in practice, concordant DZ twins can be replaced by concordant sibling pairs making sample collection easier and more feasible. On the other hand, when laboratory cost for genotyping is a major concern (such as genome-wide analysis or next-generation sequencing), MZ cases are the best choice as they help to maintain good power but with the lowest sample sizes.

Another interesting finding is the power difference between the two age cutoffs. For both MZ and DZ twins, approximately 2 to 3 times larger sample sizes are needed for cases of 90+ as compared with cases of 95+. For example, for an additive effect allele with MAF = 0.5 and *r* = 0.9, the power for 300 cases of 95+ is equivalent to that for 800 cases of 90+ in both Tables 1 and 2. The large difference in power is understandable considering the very high selection pressure going on during this age interval. Survival data from the Danish 1905 birth cohort showed equal chances for surviving from birth to age 92 as from age 92 to 100 [11]. As a trade-off for power advantage, the extremely high survival selection also adds difficulty in collecting concordant twin pairs aged 95+. The study design should always balance sampling feasibility, power, and age cutoff.

The power advantage in using singleton cases from longevity concordant twin pairs is purely due to increased likelihood for carrying longevity-linked genetic variants. For the same sample size, this study design has the same genotyping cost as an ordinary case-control study but with much higher power. In other words, acceptable power can be achieved with lower cost. This is especially important as current techniques for genomic analysis, for example, the microarray and the next-generation sequencing techniques, are still expensive. Although this study focuses only on power advantage of using twins in longevity studies, our results should also reflect similar situation in human disease studies, that is, using disease concordant twins as cases. Moreover, although our current study focuses on advantage of concordant MZ twins in gene-longevity association studies, MZ twin pairs discordant for longevity or diseases are also useful samples for looking for environmental factors. Taking all together, the unique samples of twins will have a good potential in contributing to the molecular genetic studies of human complex diseases and traits.

## Figures and Tables

**Table 1:** Power estimates for additive effects using longevity concordant MZ twins.

Relative risk	90+					95+				
	100	200	300	500	800	100	200	300	500	800
MAF = 0.05										
0.5	0.95	1.00	1.00	1.00	1.00	1.00	1.00	1.00	1.00	1.00
0.7	0.46	0.78	0.90	0.99	1.00	0.94	1.00	1.00	1.00	1.00
0.8	0.21	0.40	0.53	0.75	0.92	0.54	0.84	0.95	1.00	1.00
0.85	0.13	0.22	0.32	0.49	0.69	0.31	0.58	0.72	0.93	0.99
0.9	0.09	0.12	0.17	0.23	0.34	0.14	0.29	0.35	0.59	0.74
0.95	0.06	0.06	0.08	0.10	0.13	0.06	0.10	0.12	0.19	0.25
MAF = 0.1										
0.5	1.00	1.00	1.00	1.00	1.00	1.00	1.00	1.00	1.00	1.00
0.7	0.73	0.95	0.99	1.00	1.00	0.99	1.00	1.00	1.00	1.00
0.8	0.37	0.62	0.80	0.96	1.00	0.80	0.98	1.00	1.00	1.00
0.85	0.21	0.39	0.52	0.76	0.92	0.51	0.83	0.94	0.99	1.00
0.9	0.11	0.19	0.27	0.41	0.56	0.25	0.44	0.59	0.81	0.96
0.95	0.06	0.07	0.11	0.13	0.19	0.08	0.14	0.17	0.30	0.38
MAF = 0.2										
0.5	1.00	1.00	1.00	1.00	1.00	1.00	1.00	1.00	1.00	1.00
0.7	0.92	1.00	1.00	1.00	1.00	1.00	1.00	1.00	1.00	1.00
0.8	0.58	0.87	0.96	1.00	1.00	0.96	1.00	1.00	1.00	1.00
0.85	0.34	0.61	0.77	0.94	0.99	0.78	0.96	1.00	1.00	1.00
0.9	0.19	0.32	0.42	0.61	0.81	0.43	0.66	0.84	0.96	1.00
0.95	0.09	0.09	0.13	0.21	0.30	0.13	0.20	0.29	0.46	0.62
MAF = 0.5										
0.5	1.00	1.00	1.00	1.00	1.00	1.00	1.00	1.00	1.00	1.00
0.7	0.98	1.00	1.00	1.00	1.00	1.00	1.00	1.00	1.00	1.00
0.8	0.74	0.96	0.99	1.00	1.00	0.99	1.00	1.00	1.00	1.00
0.85	0.48	0.79	0.91	0.99	1.00	0.87	0.99	1.00	1.00	1.00
0.9	0.22	0.43	0.57	0.81	0.95	0.53	0.82	0.95	1.00	1.00
0.95	0.08	0.13	0.17	0.25	0.41	0.16	0.30	0.40	0.61	0.82

Power estimates over the lines are acceptable.

**Table 2:** Power estimates for dominant effects using longevity concordant MZ twins.

Relative risk	90+					95+				
	100	200	300	500	800	100	200	300	500	800
MAF = 0.05										
0.5	0.93	1.00	1.00	1.00	1.00	1.00	1.00	1.00	1.00	1.00
0.7	0.45	0.73	0.89	0.98	1.00	0.91	1.00	1.00	1.00	1.00
0.8	0.21	0.38	0.51	0.72	0.90	0.53	0.81	0.94	0.99	1.00
0.85	0.14	0.22	0.30	0.46	0.64	0.30	0.51	0.69	0.90	0.98
0.9	0.08	0.12	0.15	0.23	0.32	0.16	0.23	0.36	0.56	0.71
0.95	0.06	0.07	0.07	0.09	0.12	0.07	0.09	0.12	0.15	0.22
MAF = 0.1										
0.5	1.00	1.00	1.00	1.00	1.00	1.00	1.00	1.00	1.00	1.00
0.7	0.67	0.93	0.99	1.00	1.00	0.99	1.00	1.00	1.00	1.00
0.8	0.34	0.57	0.74	0.93	0.99	0.74	0.96	0.99	1.00	1.00
0.85	0.19	0.33	0.46	0.70	0.88	0.47	0.76	0.89	0.99	1.00
0.9	0.11	0.17	0.24	0.34	0.50	0.22	0.38	0.51	0.75	0.93
0.95	0.06	0.08	0.09	0.11	0.16	0.07	0.12	0.17	0.26	0.35
MAF = 0.2										
0.5	1.00	1.00	1.00	1.00	1.00	1.00	1.00	1.00	1.00	1.00
0.7	0.84	0.98	1.00	1.00	1.00	1.00	1.00	1.00	1.00	1.00
0.8	0.46	0.74	0.90	0.99	1.00	0.85	0.99	1.00	1.00	1.00
0.85	0.27	0.48	0.62	0.85	0.97	0.61	0.89	0.97	1.00	1.00
0.9	0.14	0.22	0.32	0.49	0.68	0.32	0.51	0.69	0.89	0.97
0.95	0.08	0.09	0.11	0.15	0.22	0.10	0.16	0.22	0.33	0.46
MAF = 0.5										
0.5	1.00	1.00	1.00	1.00	1.00	1.00	1.00	1.00	1.00	1.00
0.7	0.76	0.96	1.00	1.00	1.00	0.98	1.00	1.00	1.00	1.00
0.8	0.38	0.64	0.83	0.97	1.00	0.74	0.95	0.99	1.00	1.00
0.85	0.23	0.41	0.56	0.78	0.93	0.48	0.75	0.91	0.99	1.00
0.9	0.11	0.19	0.28	0.43	0.61	0.25	0.43	0.58	0.81	0.94
0.95	0.06	0.08	0.10	0.13	0.18	0.10	0.13	0.18	0.32	0.42

Power estimates over the lines are acceptable.

**Table 3:** Power estimates for recessive effects using longevity concordant MZ twins.

Relative risk	90+					95+				
	100	200	300	500	800	100	200	300	500	800
MAF = 0.05										
0.5	—	0.07	0.15	0.26	0.41	0.15	0.47	0.66	0.87	0.99
0.7	—	—	0.05	0.10	0.12	—	0.06	0.15	0.26	0.38
0.8	—	—	—	—	0.08	—	—	—	0.10	0.13
0.85	—	—	—	—	0.07	—	—	—	0.07	0.10
0.9	—	—	—	—	0.06	—	—	—	—	0.08
0.95	—	—	—	—	—	—	—	—	—	0.05
MAF = 0.1										
0.5	0.25	0.48	0.70	0.88	0.98	0.85	1.00	1.00	1.00	1.00
0.7	0.07	0.13	0.20	0.31	0.47	0.18	0.40	0.57	0.81	0.96
0.8	—	0.08	0.10	0.12	0.19	0.09	0.13	0.23	0.31	0.52
0.85	—	0.06	0.08	0.09	0.12	—	0.11	0.11	0.18	0.30
0.9	—	0.05	0.05	0.05	0.07	—	0.07	0.08	0.08	0.15
0.95	—	—	0.05	—	0.06	—	0.05	—	0.07	0.08
MAF = 0.2										
0.5	0.90	1.00	1.00	1.00	1.00	1.00	1.00	1.00	1.00	1.00
0.7	0.29	0.50	0.68	0.88	0.98	0.75	0.96	1.00	1.00	1.00
0.8	0.13	0.20	0.29	0.45	0.66	0.32	0.55	0.75	0.91	0.98
0.85	0.08	0.12	0.17	0.26	0.38	0.17	0.31	0.40	0.63	0.85
0.9	0.06	0.07	0.10	0.13	0.18	0.08	0.15	0.17	0.26	0.41
0.95	0.05	0.06	0.07	0.06	0.09	0.05	0.09	0.08	0.08	0.12
MAF = 0.5										
0.5	1.00	1.00	1.00	1.00	1.00	1.00	1.00	1.00	1.00	1.00
0.7	0.92	1.00	1.00	1.00	1.00	1.00	1.00	1.00	1.00	1.00
0.8	0.51	0.77	0.91	0.99	1.00	0.90	1.00	1.00	1.00	1.00
0.85	0.28	0.47	0.65	0.87	0.97	0.63	0.89	0.98	1.00	1.00
0.9	0.14	0.22	0.31	0.45	0.64	0.26	0.50	0.68	0.87	0.98
0.95	0.06	0.08	0.11	0.14	0.19	0.09	0.16	0.20	0.30	0.46

^a^Power estimates over the lines are acceptable.

^b^Dashes indicate power estimates <0.05.

**Table 4:** Power estimates for additive effects using longevity concordant DZ twins.

Relative risk	90+						95+					
	100	200	300	500	800	1500	100	200	300	500	800	1500
MAF = 0.05												
0.5	0.73	0.94	0.98	1.00	1.00	1.00	1.00	1.00	1.00	1.00	1.00	1.00
0.7	0.30	0.53	0.71	0.87	0.97	1.00	0.74	0.95	0.99	1.00	1.00	1.00
0.8	0.16	0.23	0.34	0.51	0.72	0.92	0.34	0.56	0.73	0.93	0.99	1.00
0.85	0.10	0.16	0.17	0.31	0.44	0.69	0.20	0.34	0.47	0.70	0.89	0.99
0.9	0.07	0.08	0.12	0.14	0.24	0.37	0.12	0.19	0.25	0.37	0.50	0.77
0.95	0.06	0.05	0.06	0.08	0.09	0.11	0.08	0.08	0.08	0.11	0.14	0.24
MAF = 0.1												
0.5	0.92	0.99	1.00	1.00	1.00	1.00	1.00	1.00	1.00	1.00	1.00	1.00
0.7	0.50	0.74	0.90	0.99	1.00	1.00	0.93	0.99	1.00	1.00	1.00	1.00
0.8	0.25	0.44	0.54	0.76	0.92	0.99	0.57	0.83	0.95	1.00	1.00	1.00
0.85	0.15	0.24	0.32	0.48	0.68	0.90	0.32	0.56	0.74	0.92	0.99	1.00
0.9	0.09	0.13	0.17	0.26	0.35	0.64	0.16	0.28	0.36	0.55	0.75	0.96
0.95	0.06	0.06	0.07	0.09	0.12	0.19	0.06	0.11	0.13	0.16	0.26	0.43
MAF = 0.2												
0.5	0.97	1.00	1.00	1.00	1.00	1.00	1.00	1.00	1.00	1.00	1.00	1.00
0.7	0.68	0.93	0.99	1.00	1.00	1.00	0.99	1.00	1.00	1.00	1.00	1.00
0.8	0.36	0.63	0.79	0.94	0.99	1.00	0.78	0.98	1.00	1.00	1.00	1.00
0.85	0.24	0.35	0.53	0.75	0.92	1.00	0.51	0.81	0.94	0.99	1.00	1.00
0.9	0.12	0.21	0.29	0.39	0.59	0.84	0.23	0.45	0.61	0.80	0.94	1.00
0.95	0.06	0.08	0.12	0.13	0.19	0.31	0.09	0.14	0.17	0.25	0.44	0.68
MAF = 0.5												
0.5	1.00	1.00	1.00	1.00	1.00	1.00	1.00	1.00	1.00	1.00	1.00	1.00
0.7	0.84	0.98	1.00	1.00	1.00	1.00	1.00	1.00	1.00	1.00	1.00	1.00
0.8	0.48	0.79	0.91	0.99	1.00	1.00	0.88	1.00	1.00	1.00	1.00	1.00
0.85	0.30	0.55	0.71	0.88	0.98	1.00	0.67	0.90	0.98	1.00	1.00	1.00
0.9	0.16	0.25	0.38	0.57	0.72	0.95	0.33	0.56	0.74	0.92	0.99	1.00
0.95	0.08	0.09	0.12	0.18	0.25	0.41	0.12	0.18	0.26	0.40	0.58	0.83

^a^Power estimates over the lines are acceptable.

**Table 5:** Power estimates for dominant effects using longevity concordant DZ twins.

Relative risk	90+						95+					
	100	200	300	500	800	1500	100	200	300	500	800	1500
MAF = 0.05												
0.5	0.72	0.93	0.99	1.00	1.00	1.00	1.00	1.00	1.00	1.00	1.00	1.00
0.7	0.28	0.47	0.65	0.85	0.97	1.00	0.64	0.91	0.99	1.00	1.00	1.00
0.8	0.15	0.24	0.32	0.48	0.68	0.92	0.32	0.56	0.72	0.91	0.99	1.00
0.85	0.10	0.16	0.16	0.28	0.40	0.66	0.20	0.34	0.45	0.63	0.84	0.98
0.9	0.07	0.08	0.12	0.14	0.20	0.34	0.12	0.16	0.21	0.33	0.49	0.75
0.95	0.05	0.06	0.05	0.07	0.09	0.12	0.07	0.07	0.08	0.11	0.14	0.24
MAF = 0.1												
0.5	0.88	0.99	1.00	1.00	1.00	1.00	1.00	1.00	1.00	1.00	1.00	1.00
0.7	0.44	0.71	0.84	0.98	1.00	1.00	0.85	0.99	1.00	1.00	1.00	1.00
0.8	0.18	0.37	0.49	0.69	0.88	0.99	0.49	0.76	0.91	0.98	1.00	1.00
0.85	0.12	0.19	0.30	0.47	0.63	0.88	0.28	0.51	0.69	0.88	0.98	1.00
0.9	0.09	0.14	0.15	0.22	0.33	0.55	0.15	0.23	0.35	0.51	0.70	0.92
0.95	0.06	0.05	0.06	0.07	0.11	0.16	0.07	0.09	0.10	0.15	0.22	0.36
MAF = 0.2												
0.5	0.97	1.00	1.00	1.00	1.00	1.00	1.00	1.00	1.00	1.00	1.00	1.00
0.7	0.58	0.84	0.95	1.00	1.00	1.00	0.94	1.00	1.00	1.00	1.00	1.00
0.8	0.28	0.50	0.65	0.86	0.96	1.00	0.64	0.90	0.98	1.00	1.00	1.00
0.85	0.17	0.29	0.36	0.58	0.80	0.97	0.41	0.62	0.80	0.95	0.99	1.00
0.9	0.12	0.14	0.21	0.28	0.43	0.69	0.19	0.31	0.47	0.65	0.84	0.98
0.95	0.06	0.07	0.08	0.10	0.11	0.20	0.08	0.10	0.15	0.21	0.31	0.51
MAF = 0.5												
0.5	0.95	1.00	1.00	1.00	1.00	1.00	1.00	1.00	1.00	1.00	1.00	1.00
0.7	0.46	0.77	0.90	0.98	1.00	1.00	0.84	0.98	1.00	1.00	1.00	1.00
0.8	0.25	0.41	0.54	0.76	0.91	1.00	0.46	0.73	0.89	0.99	1.00	1.00
0.85	0.15	0.24	0.34	0.49	0.69	0.90	0.28	0.49	0.65	0.86	0.98	1.00
0.9	0.08	0.12	0.16	0.25	0.35	0.58	0.14	0.25	0.32	0.54	0.70	0.93
0.95	0.05	0.07	0.08	0.09	0.13	0.17	0.06	0.10	0.11	0.17	0.24	0.40

^a^Power estimates over the lines are acceptable.

**Table 6:** Power estimates for recessive effects using longevity concordant DZ twins.

Relative risk	90+						95+					
	100	200	300	500	800	1500	100	200	300	500	800	1500
MAF = 0.05												
0.5	—	—	0.07	0.13	0.19	0.32	—	0.14	0.26	0.42	0.60	0.89
0.7	—	—	—	0.05	0.08	0.09	—	—	0.05	0.11	0.15	0.28
0.8	—	—	—	—	0.06	0.07	—	—	—	0.06	0.09	0.12
0.85	—	—	—	—	0.05	0.07	—	—	—	—	0.06	0.07
0.9	—	—	—	—	—	—	—	—	—	—	0.05	0.05
0.95	—	—	—	—	0.05	—	—	—	—	—	—	0.05
MAF = 0.1												
0.5	0.11	0.18	0.32	0.46	0.68	0.91	0.45	0.73	0.91	0.99	1.00	1.00
0.7	0.05	0.07	0.09	0.13	0.23	0.36	0.08	0.17	0.25	0.36	0.57	0.83
0.8	—	0.06	0.07	0.09	0.11	0.16	—	0.07	0.09	0.15	0.26	0.41
0.85	—	0.05	0.06	0.06	0.09	0.09	—	0.07	0.09	0.11	0.14	0.24
0.9	—	—	0.06	—	0.06	0.09	—	0.05	—	0.06	0.08	0.10
0.95	—	—	0.05	0.05	0.05	0.05	—	—	0.05	—	0.06	0.06
MAF = 0.2												
0.5	0.51	0.77	0.92	0.99	1.00	1.00	0.99	1.00	1.00	1.00	1.00	1.00
0.7	0.14	0.23	0.36	0.51	0.73	0.93	0.38	0.62	0.82	0.95	1.00	1.00
0.8	0.08	0.12	0.16	0.21	0.33	0.57	0.14	0.24	0.34	0.56	0.76	0.96
0.85	—	0.07	0.10	0.15	0.18	0.30	0.08	0.14	0.19	0.30	0.46	0.71
0.9	0.06	0.06	0.08	0.07	0.11	0.13	0.06	0.08	0.12	0.14	0.20	0.35
0.95	—	0.05	0.05	0.05	0.05	0.08	0.05	0.06	0.06	0.08	0.08	0.10
MAF = 0.5												
0.5	1.00	1.00	1.00	1.00	1.00	1.00	1.00	1.00	1.00	1.00	1.00	1.00
0.7	0.68	0.90	0.99	1.00	1.00	1.00	0.98	1.00	1.00	1.00	1.00	1.00
0.8	0.28	0.49	0.65	0.86	0.96	1.00	0.64	0.90	0.98	1.00	1.00	1.00
0.85	0.16	0.26	0.38	0.57	0.75	0.95	0.35	0.62	0.78	0.94	0.99	1.00
0.9	0.09	0.12	0.20	0.27	0.39	0.62	0.16	0.27	0.38	0.56	0.76	0.97
0.95	0.07	0.07	0.08	0.09	0.12	0.17	0.08	0.09	0.12	0.18	0.27	0.44

^a^Power estimates over the lines are acceptable.

^b^Dashes indicate power estimates <0.05.
